# Exploring Neurodegenerative Risk Genes in ALS

**DOI:** 10.1002/alz70855_107327

**Published:** 2025-12-24

**Authors:** Mari J Tokita, Justin Y Kwan, Andrew J Oler, Laura E Danielian, Jody Crook, Kateyln Porter, Magdalena A Walkiewicz, Allison Snyder

**Affiliations:** ^1^ Centralized sequencing program, National Institute of Allergy and Infectious Diseases, Bethesda, MD, USA; ^2^ National Institute of Neurological Disorders and Stroke, Bethesda, MD, USA; ^3^ Bioinformatics and Computational Biosciences Branch, Office of Cyber Infrastructure and Computational Biology, National Institute of Allergy and Infectious Diseases, Bethesda, MD, USA; ^4^ Penn Frontotemporal Degeneration Center, Department of Neurology, Perelman School of Medicine, University of Pennsylvania, Philadelphia, PA, USA

## Abstract

**Background:**

*APOE* and *TMEM106B* genotypes and *MAPT* haplotypes are established risk factors for neurodegenerative disease. There is conflicting evidence in the literature regarding the association of these risk factors with amyotrophic lateral sclerosis (ALS). We aimed to perform a systematic analysis of all three risk factors in an ALS cohort to understand their prevalence and to identify associations with disease outcomes in comparison to a broader neurodegenerative cohort.

**Method:**

Participants evaluated at the NIH Neurodegenerative Disorders Clinic were included in the analysis. Whole genome sequencing was performed to a mean coverage of 30x. Genome data were queried for high penetrance disease‐associated variants, and the following SNPs were genotyped for *MAPT* haplotyping (rs8070723) and to determine *APOE* (rs429358 and rs7412) and *TMEM106B* risk allele status (rs1990622).

**Result:**

This preliminary analysis included 34 study participants of whom 53% were male; mean age was 64.1 years +/‐ 12.3. 21% of participants (*n* = 7) carried a diagnosis of ALS and the remaining 79% (*n* = 27) had non‐ALS neurodegenerative disease (FTD, AD, DLB). The average CDR NACC FTLD‐M sum of boxes was 5.8 +/‐ 6.5. One autosomal dominant pathogenic variant was identified in each group. Heterozygosity for *APOE* e4 was detected in 28.6% of ALS and 48.1% of non‐ALS participants; 0 ALS and 11.1% of non‐ALS participants were heterozygous for *APOE* e2. 71.4% of ALS and 81.5% of non‐ALS participants harbored at least one *TMEM106B* risk allele. Similarly, 71.4% of ALS and 81.5% of non‐ALS subjects were homozygous for the *MAPT* H1 haplotype.

**Conclusion:**

While the differences between cohorts did not meet statistical significance (likely due to small sample sizes) the observed patterns are consistent with known associations of the risk loci. *APOE* e4 genetic risk was not enriched in the ALS cohort. *TMEM106B*‐mediated risk and, unexpectedly given its association with tauopathies, *MAPT* H1 haplotype status were similar in both ALS and non‐ALS cohorts. Future analyses will focus on increasing our sample size and defining the relationship of risk alleles to disease characteristics.

